# Systematically Altering Bacterial SOS Activity under Stress Reveals Therapeutic Strategies for Potentiating Antibiotics

**DOI:** 10.1128/mSphere.00163-16

**Published:** 2016-08-10

**Authors:** Charlie Y. Mo, Sara A. Manning, Manuela Roggiani, Matthew J. Culyba, Amanda N. Samuels, Paul D. Sniegowski, Mark Goulian, Rahul M. Kohli

**Affiliations:** a Biochemistry and Molecular Biophysics Graduate Group, Perelman School of Medicine at the University of Pennsylvania, Philadelphia, Pennsylvania, USA; b Department of Medicine, Perelman School of Medicine at the University of Pennsylvania, Philadelphia, Pennsylvania, USA; c Department of Biochemistry and Biophysics, Perelman School of Medicine at the University of Pennsylvania, Philadelphia, Pennsylvania, USA; d Department of Biology, School of Arts and Sciences, University of Pennsylvania, Philadelphia, Pennsylvania, USA; University of Rochester

**Keywords:** DNA damage, LexA, RecA, SOS pathway, adjuvant therapy, antibiotic resistance, mutagenesis

## Abstract

The bacterial SOS response is a DNA damage repair network that is strongly implicated in both survival and acquired drug resistance under antimicrobial stress. The two SOS regulators, LexA and RecA, have therefore emerged as potential targets for adjuvant therapies aimed at combating resistance, although many open questions remain. For example, it is not well understood whether SOS hyperactivation is a viable therapeutic approach or whether LexA or RecA is a better target. Furthermore, it is important to determine which antimicrobials could serve as the best treatment partners with SOS-targeting adjuvants. Here we derived *Escherichia coli* strains that have mutations in either *lexA or recA* genes in order to cover the full spectrum of possible SOS activity levels. We then systematically analyzed a wide range of antimicrobials by comparing the mean inhibitory concentrations (MICs) and induced mutation rates for each drug-strain combination. We first show that significant changes in MICs are largely confined to DNA-damaging antibiotics, with strains containing a constitutively repressed SOS response impacted to a greater extent than hyperactivated strains. Second, antibiotic-induced mutation rates were suppressed when SOS activity was reduced, and this trend was observed across a wider spectrum of antibiotics. Finally, perturbing either LexA or RecA proved to be equally viable strategies for targeting the SOS response. Our work provides support for multiple adjuvant strategies, while also suggesting that the combination of an SOS inhibitor with a DNA-damaging antibiotic could offer the best potential for lowering MICs and decreasing acquired drug resistance.

**IMPORTANCE** Our antibiotic arsenal is becoming depleted, in part, because bacteria have the ability to rapidly adapt and acquire resistance to our best agents. The SOS pathway, a widely conserved DNA damage stress response in bacteria, is activated by many antibiotics and has been shown to play central role in promoting survival and the evolution of resistance under antibiotic stress. As a result, targeting the SOS response has been proposed as an adjuvant strategy to revitalize our current antibiotic arsenal. However, the optimal molecular targets and partner antibiotics for such an approach remain unclear. In this study, focusing on the two key regulators of the SOS response, LexA and RecA, we provide the first comprehensive assessment of how to target the SOS response in order to increase bacterial susceptibility and reduce mutagenesis under antibiotic treatment.

## Abstract (precis)

Our antibiotic arsenal is becoming depleted, in part, because bacteria have the ability to rapidly adapt and acquire resistance to our best agents. The SOS pathway, a widely conserved DNA damage stress response in bacteria, is activated by many antibiotics and has been shown to play central role in promoting survival and the evolution of resistance under antibiotic stress. As a result, targeting the SOS response has been proposed as an adjuvant strategy to revitalize our current antibiotic arsenal. However, the optimal molecular targets and partner antibiotics for such an approach remain unclear. In this study, focusing on the two key regulators of the SOS response, LexA and RecA, we provide the first comprehensive assessment of how to target the SOS response in order to increase bacterial susceptibility and reduce mutagenesis under antibiotic treatment.

## INTRODUCTION

The rapid rise of antibiotic resistance in bacterial pathogens is a major global health crisis. In the United States alone, resistant bacteria are associated with approximately 2 million infections and 23,000 deaths per year, with an economic burden upwards of $55 billion (1). In the past decade, the frequency of multidrug-resistant strains has rapidly risen, and even sensitive organisms are requiring higher MICs for effective therapy (2, 3). Efforts to address the problem of resistance have traditionally focused on modifying the scaffolds of existing antibiotics in order to circumvent the molecular mechanisms conferring resistance (4). While such efforts can offer a respite, resistance often rapidly follows, as existing resistance determinants adapt to the new agents (5). As a result, alternative strategies are being pursued, such as mining for previously inaccessible natural products, potentiating the host immune response, and targeting bacterial virulence pathways (6–10).

One of the new strategies proposed to combat resistance is to target the bacterial DNA damage stress response pathway, also known as the SOS response (11–14). The SOS response is a widely conserved DNA damage repair network that enables bacteria to survive genotoxic damage, but is also strongly associated with elevated mutagenesis and acquired resistance (15–17). The SOS pathway consists of a set of genes (SOS genes), which are defined to be under the control of the RecA and LexA proteins (18, 19). LexA is a dual-function repressor protease that blocks the transcription of the SOS genes in the absence of stress. When bacteria experience genotoxic stress, RecA, acting as a sensor molecule, polymerizes along exposed single-stranded DNA (ssDNA), forming activated nucleoprotein filaments (RecA*). RecA*, in turn, stimulates LexA to undergo autoproteolysis (self-cleavage), leading to the derepression of SOS effector genes.

The induced SOS effector genes can play roles in adaptation to antibiotic stress, acquired resistance, or pathogenicity. One such effector, *sulA*, encodes an inhibitor of cell division, which has been proposed to serve as a DNA damage checkpoint during the response (20). Other SOS effectors facilitate high-fidelity DNA damage repair, such as *uvrA*, which is involved in nucleotide excision repair (21), and *recA* itself, which participates in homologous recombination (22). However, under higher levels of damage, lower-fidelity processes emerge and can predominate in the response. Chief among these error-prone SOS effectors are *umuDC* and *dinB*, which encode translesion DNA polymerases that are able to replicate over genomic lesions, but do so at the expense of increased mutagenesis (17, 23). In addition, SOS activation has also been shown to trigger the expression of integrases that mediate transfer of resistance genes. On a phenotypic level, the response is also implicated in biofilm formation, induction of persister states, and expression of virulence factors (24–27). Thus, SOS activation serves to promote DNA damage tolerance and survival under genotoxic stress, while also increasing the likelihood of acquiring antibiotic resistance.

Many antibiotics trigger the SOS response, either directly through DNA damage (e.g., fluoroquinolones) or indirectly through alternative activation pathways (e.g., β-lactams) (28–30). Given its role in adaptation and acquired resistance, the SOS response has therefore been proposed as an attractive therapeutic target that might potentiate our current antibiotic arsenal. Various lines of genetic evidence support this possibility. Extensive historical studies have shown that mutations in *recA* and *lexA* can increase bacterial sensitivity to DNA-damaging agents such as UV radiation (31–33), and these findings also extend to medically relevant antibiotics. For example, in a murine thigh infection model, Cirz et al. demonstrated that inactivating LexA autoproteolysis reduces both the viability and acquired resistance of *Escherichia coli* treated with either ciprofloxacin or rifampin (12); likewise, Thi et al. showed that *E. coli* strains with *recA* deleted exhibit increased antibiotic sensitivity and reduced mutagenesis under a wide range of drug treatments (34).

The ramifications of hyperactivating the SOS response are less well understood, but could also offer potential therapeutic avenues. Early work on *E. coli* with a mutant *lexA* gene that resulted in a constitutive SOS activation showed heightened resistance to UV radiation and elevated mutation levels (35, 36). However, the effects of constitutive SOS activation on antibiotic susceptibility remain, to our knowledge, less well defined. Since the SOS response is part of a complex network of genes (37, 38), an overactive response could disrupt coordination of DNA damage repair and increase sensitivity to antimicrobials. Further, increased expression of some SOS effectors could enhance the effect of some antibiotics. For example, in *E. coli* and other bacterial species, deletion of the *lexA* gene is lethal to the cell, because constitutive expression of *sulA* permanently arrests cell division (20, 39). Additionally, a higher mutagenic burden associated with expression of error-prone SOS effectors could compromise fitness, analogous to lethal mutagenesis strategies utilized to combat some viruses (40).

**FIG 1:**
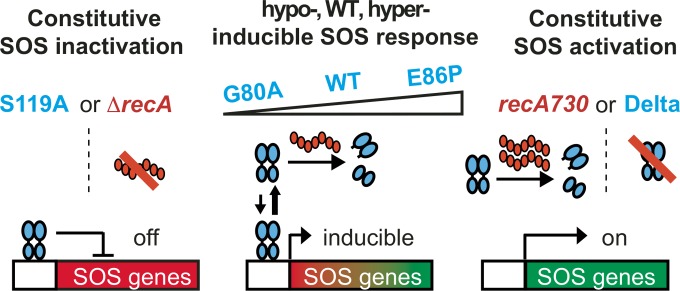
Engineered *lexA* and *recA* variants in *E. coli* displaying a range of SOS activities. The LexA protein is represented by blue ovals, and the various LexA cleavage mutants are labeled in blue. RecA is shown as red ovals, and variants are labeled in red. Five LexA variants and two RecA variants allow for examination of the spectrum of SOS activation. Activated RecA filaments lead to cleavage of LexA and inducible expression of SOS genes in the WT strain. Inactivation of LexA self-cleavage (S119A) or deletion of *recA* (Δ*recA*) inactivates the response. Mutations in the LexA protein can either decrease (G80A) or increase (E86P) the rate of self-cleavage relative to the WT strain and thus affect the level of SOS induction. Deletion of *lexA* (Delta) or hyperactivation of RecA (*recA730*) leads to constitutive expression of SOS genes.

Despite the strong genetic data implicating the SOS response as critical for survival and adaption of bacteria under stress, significant questions remain regarding targeting of the SOS response. What is the best strategy for perturbing the SOS regulatory network, and which antibiotics would serve as the best partners for SOS-targeting adjuvants? What is the relative viability of targeting RecA versus LexA? What are the implications of hyperactivating versus inhibiting the SOS pathway? To address these questions, we generated *E. coli* mutants that exhibit a spectrum of SOS activities, ranging from constitutively repressed to constitutively active (Fig. 1). These strains provided us with the tools to systematically measure bacterial susceptibility and induced mutation rates to different classes of antibiotics. Our comprehensive analysis offers guidance for strategies to combat drug resistance by targeting the SOS response.

## RESULTS

### Generation of *recA* and *lexA* mutant strains in *E. coli.*

While isolated studies using strains with inactivated *lexA* or deleted *recA* have validated the SOS pathway as a therapeutic target, a systematic comparison across the entire spectrum of SOS activity could help guide therapeutic strategies. Toward this goal, we derived a series of MG1655 *E. coli* strains that enabled us to systematically examine the consequences of altered SOS activity. We aimed to engineer strains with *lexA* variations that would span from a constitutively inactive to a constitutively active SOS response and also mimic the extreme phenotypes with alterations to *recA* (Fig. 1).

As deletion of *lexA* is lethal in *sulA^+^ E. coli* strains, we generated congenic strains in the Δ*sulA* background that only differ in the proficiency of the LexA protein to undergo self-cleavage. To access one extreme, the constitutively active SOS pathway, we first constructed a Δ*lexA* strain in the SulA-null background (defined as the Delta strain). This Delta strain was subsequently used as the parent for genetic recombineering at the *lexA* locus to yield four previously described LexA cleavage rate variants: a “hyperactive” E86P mutant, the reconstituted wild-type (WT) enzyme, a “hypoactive” G80A mutant, and finally the SOS-inactive S119A mutant, which is completely incompetent in self-cleavage (41, 42). The relative *in vitro* self-cleavage activities of these LexA variants were confirmed in biochemical assays, with G80A ~5-fold reduced and E86P ~10-fold enhanced relative to WT LexA and with S119A unable to self-cleave under both base- and RecA-mediated conditions (see Fig. S1 in the supplemental material). Together these five congenic strains provide the full spectrum of SOS activities, spanning from constitutively repressed (S119A) to constitutively active (Delta).

10.1128/mSphere.00163-16.2 Figure S1  LexA mutants display a spectrum of cleavage activities. (A) Base-mediated cleavage activity of S119A (red), G80A (gray), WT (blue), and E86P (purple) LexA. The fraction of full-length protein remaining over time was fit to a first-order exponential decay, and the rate constant (*k*) was calculated. (B) RecA*-induced LexA cleavage. The left panel shows a RecA* titration cleavage assay of the mutants (0.3 µM ^32^P-labeled S119A, G80A, and WT LexA, and 2.5 µM of unlabeled E86P LexA). The right panel shows a reaction time course of the four mutants under 2.5 µM RecA* stimulation. The formation of free NTD (product) was monitored over time. The data symbol indicates the mean of 2 replicates, and the error bars denote the range of the observations. Download Figure S1, PDF file, 0.4 MB. Copyright © 2016 Mo et al. 2016 Mo et al. This content is distributed under the terms of the Creative Commons Attribution 4.0 International license.

We also sought to simultaneously compare the effect of perturbing *lexA* with alterations to *recA*. For comparison to Delta, we generated a congenic strain containing the *recA730* allele, which encodes a DNA recombination and coprotease-proficient RecA variant, the E38K strain, which forms nucleoprotein filaments in the absence of DNA damage and constitutively activates the SOS response through constant LexA cleavage (41, 43). For comparison to the S119A strain, we used a strain containing a *recA* deletion. Together, these *recA* mutants, along with the *lexA* mutants described above, are the main focus in our subsequent MIC, fitness, and mutagenesis experiments. However, we recognized that the *sulA* deletion in the Delta strain could in principle impact bacterial susceptibility and mutagenesis. We therefore also restored *sulA* in the S119A, G80A, and E86P variants to allow for comparison to the reference MG1655 strain (summarized in Table 1).

**TABLE 1:** List of *E. coli* strains used in this study

Strain	Relevant genotype	Description	Source or reference
MG1655	*lexA*^+^ *sulA*^+^	Used as WT LexA and *sulA*^+^ control in MIC and fluctuation analyses	Yale E. coli Genetic Stock Center CGSC 7740
JW0941	Δ*sulA*::(FRT*-kan-*FRT)	Strain source of Δ*sulA*	Keio collection (70)
SAMP02	MG1655 Δ*sulA*::FRT	Parent strain for Δ*lexA* strain	This study
JW3470	Δ*recA*::(FRT*-kan-*FRT)	Strain source of Δ*recA*	Keio collection (70)
Δ*recA* variant	MG1655 Δ*recA*::FRT	Used as the *recA* knockout strain	This study
Delta variant (SAMP04)	MG1655 Δ*lexA*::(Cm-I-SceI) Δ*sulA*::FRT	Parent strain for recombineering; *lexA* knockout	This study
WT	MG1655 *lexA*^+^ Δ*sulA*::FRT	Strain with WT LexA cleavage	This study
S119A variant	MG1655 *lexA*(S119A) Δ*sulA*::FRT	Strain with catalytically inactive LexA	This study
G80A variant	MG1655 *lexA*(G80A) Δ*sulA*::FRT	Strain with slow-cleaving LexA	This study
E86P variant	MG1655 *lexA*(E86P) Δ*sulA*::FRT	Strain with fast-cleaving LexA	This study
SS4247	TetR *recA730* *srlC300*::Tn*10*	Strain source of *recA730*	Steven J. Sandler, unpublished
*recA730* variant	MG1655 *lexA*^+^ Δ*sulA recA730* TetR *srlC300*::Tn*10*	Strain with WT LexA cleavage and constitutive RecA activity	This study
*recA730*(S119A) variant	MG1655 *lexA*(S119A) Δ*sulA recA730* TetR *srlC300*::Tn*10*	Strain with catalytically inactive LexA and constitutive RecA activity	This study
MMR102	MG1655 *sulA^+^* Δ*torT*::(FRT*-kan-*FRT)	Strain source of *sulA*	Goulian lab stock
S119A (*sulA^+^*) variant	MG1655 *lexA*(S119A) *sulA^+^* Δ*torT*::FRT	Strain with inactive LexA and *sulA*	This study
G80A (*sulA^+^*) variant	MG1655 *lexA*(G80A) *sulA^+^* Δ*torT*::FRT	Strain with slow-cleaving LexA and *sulA*	This study
E86P (*sulA^+^*) variant	MG1655 *lexA*(E86P) *sulA^+^* Δ*torT*::FRT	Strain with fast-cleaving LexA and *sulA*	This study

### MICs of the mutant strains under various antibiotic stress.

**FIG 2:**
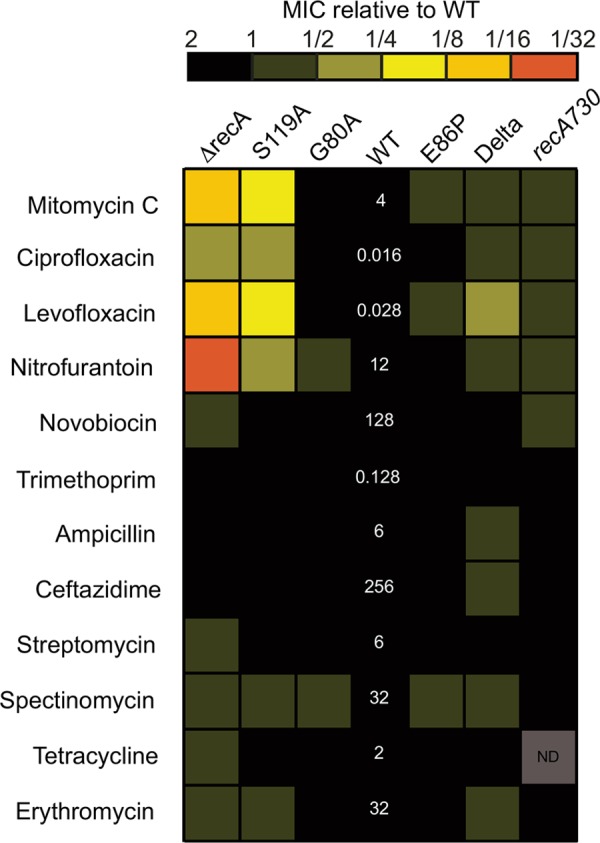
Heat map of relative MICs of different SOS variants with various antibiotics. The MIC values of the WT strain, shown in white numbers, are listed in micrograms per milliliter. MIC values represent the average from at least four independent determinations performed on separate days. “ND” represents a condition and strain in which the MIC was not determined. The raw MIC values of all strains are shown in Table S1 in the supplemental material.

With our spectrum of SOS variant strains established, we first aimed to survey their sensitivity to a broad range of different antimicrobials and DNA-damaging agents. We selected 12 drugs spanning different mechanistic classes and compared the resulting MIC for each drug-strain combination to that of the WT strain (Fig. 2; see Table S1 in the supplemental material). For mitomycin C, a DNA alkylator that is known to induce the SOS response through formation of intrastrand DNA cross-links, we observed 16-fold and 4-fold reductions of the MIC for the Δ*recA* and S119A strains, respectively. We also observed reductions in the E86P, Delta, and *recA730* strains to a lesser extent, indicating that SOS hyperactivation can also increase susceptibility to DNA damage. A similar trend in the MICs was observed with several antibiotics known to damage DNA and activate the SOS response. For the fluoroquinolones, which target DNA gyrase and induce double-stranded DNA breaks, treatment of the SOS-inactivating *lexA* and *recA* mutant strains with either ciprofloxacin or levofloxacin resulted in a ≥4-fold reduction in MIC, while the two SOS-constitutive mutants exhibited more modest decreases (between 2- and 4-fold). Finally, we observed similar susceptibility patterns with nitrofurantoin, which is associated with oxidative DNA damage (44). In comparing alterations to *lexA* versus *recA*, the sensitivities of *recA730* and Delta were generally similar to one another, while Δ*recA* had slightly enhanced sensitivity relative to the S119A strain. In particular, the Δ*recA* strain exhibits a 32-fold reduction in MIC with nitrofurantoin, the greatest change in sensitivity across strains and antibiotics evaluated.

10.1128/mSphere.00163-16.7 Table S1  MICs for drug-*E. coli* strain combinations. Download Table S1, PDF file, 0.1 MB. Copyright © 2016 Mo et al. 2016 Mo et al. This content is distributed under the terms of the Creative Commons Attribution 4.0 International license.

For the remaining antibiotics tested, we saw more modest changes in susceptibility in the strains. With novobiocin, an agent that inhibits DNA gyrase without inducing double-stranded DNA (dsDNA) breaks (45, 46), sensitivity was generally unchanged, with the exception of a 2-fold reduction in the MIC of the Δ*recA* strain. The novobiocin results suggest that the generation of dsDNA breaks by fluoroquinolines, and not gyrase inhibition alone, is needed for synergy with SOS perturbation. Although dihydrofolate reductase inhibitors, aminoglycosides, and β-lactams have been proposed to induce the SOS response, we observed ≤2-fold changes in MICs across strains. Similarly, with non-SOS-inducing antibiotics, the macrolide erythromycin, or aminoglycosides, we observed ≤2-fold changes in sensitivity at SOS extremes.

To determine whether the *sulA* deletion impacts the MIC differences, we examined antibiotic sensitivity with our corresponding *sulA*^+^ strains (which by necessity exclude the Delta and *recA730* strains). For some antibiotics, such as levofloxacin and nitrofurantoin, the MIC of the WT (*sulA*^+^) strain was reduced compared to that of the WT (Δ*sulA*) strain. However, the trend in MIC reduction between the different *sulA*^+^ strains remained consistent with the trend in Δ*sulA* SOS variants (see Fig. S2 and Table S1 in the supplemental material). Thus, we conclude that *sulA* status may alter susceptibility for a given antibiotic, but this does not change the relative impact of perturbing SOS regulation.

10.1128/mSphere.00163-16.3 Figure S2  MIC values for *sulA*-null and *sulA*^+^ strains. The left panel is a heat map of the *sulA*^+^ strains’ MICs relative to MG1655. The MIC values of the MG1655 strain are shown as white numbers in micrograms per milliliter. The right panel shows analogous data in the Δ*sulA* background, and shows a subset of the data from Fig. 2 in the main text. Download Figure S2, PDF file, 0.3 MB. Copyright © 2016 Mo et al. 2016 Mo et al. This content is distributed under the terms of the Creative Commons Attribution 4.0 International license.

### Impact of hypo- and hyperactive SOS variants on bacterial fitness.

In our MIC analysis, we noticed that only minor differences were evident between the G80A, WT, and E86P strains. We next wanted to ascertain if these intermediate SOS variants display any detectable changes in fitness to antibiotic-induced stress. We first confirmed that altering LexA cleavage activities perturbed levels of SOS induction in the predicted manner. To do this, we employed a green fluorescent protein (GFP) reporter plasmid in which *gfp* is placed under the control of the SOS-inducible *recA* promoter (47). We measured GFP fluorescence in each *lexA* variant strain in the presence of sublethal doses of mitomycin C, ciprofloxacin, or nitrofurantoin. The GFP fluorescence profiles of the strains correlated with the rate of self-cleavage for each LexA cleavage rate variant, thus confirming differences in extent of SOS activation across strains (see Fig. S3 in the supplemental material).

10.1128/mSphere.00163-16.4 Figure S3  Bacterial SOS induction directly correlates with *in vitro* LexA cleavage rates. (A) GFP fluorescence normalized by optimal density of the *lexA* strains during a 180-min exposure to no stress or sublethal doses of ciprofloxacin, mitomycin C, and nitrofurantoin. The data are represented as the means from 3 independent measurements, and the error bars reflect the standard errors from the 3 replicates. (B) Mean GFP induction under mitomycin C, nitrofurantoin, and streptomycin stress after 16 h evaluated by flow cytometry. The data bars represent the averages from two independent measurements, and error bars denote the ranges of observed values. Download Figure S3, PDF file, 0.4 MB. Copyright © 2016 Mo et al. 2016 Mo et al. This content is distributed under the terms of the Creative Commons Attribution 4.0 International license.

**FIG 3:**
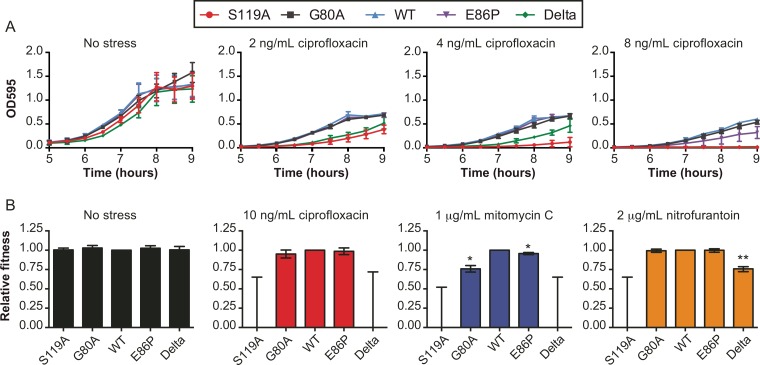
Impact of *lexA* variants on cell growth under sublethal doses of stress. (A) Growth curves of the strains exposed to increasing, yet sublethal, levels of ciprofloxacin stress. The data are represented as the mean of 3 independent measurements, and the error bars reflect the standard errors of the 3 replicates. Growth curves under mitomycin C and nitrofurantoin stress are shown in Fig. S4 in the supplemental material. (B) Estimated fitness of the variant strains relative to the WT strain under different types of antibiotic stress. The mean fitness of each strain relative to the WT strain was calculated from 3 independent competition experiments. Error bars represent the standard errors from the three trials. For strains in which no colonies were detected for the variant strain, the top of the error bar represents the limit of detection. Significant *P* values are noted (*, <0.05; **, <0.005).

Although we did not detect large changes in MICs between the intermediate SOS variant strains, we considered whether these strains might have relative growth deficits compared to one another. To this end, we measured exponential growth in the presence or absence of sublethal antibiotic stress across all five strains (Fig. 3; see Fig. S4 in the supplemental material). In the absence of any antibiotic stress, all strains displayed similar growth rates. However, when the strains were treated with ciprofloxacin at sublethal levels, growth was significantly perturbed for the S119A and Delta strains, as expected. As ciprofloxacin approached the MIC, E86P had a reduction in growth rate, while G80A remained similar to the WT (Fig. 3A). For mitomycin C and nitrofurantoin, the S119A and Delta strains were again compromised, the G80A strain showed minor growth reductions, and the E86P strain remained similar to the WT (see Fig. S4).

10.1128/mSphere.00163-16.5 Figure S4  Growth curves of variant *E. coli* stains under mitomycin C and nitrofurantoin stress. The top panel is the same no-stress growth curve shown in Fig. 3. The data are represented as the means from 3 independent measurements, and the error bars reflect the standard errors from the 3 replicates. Download Figure S4, PDF file, 0.3 MB. Copyright © 2016 Mo et al. 2016 Mo et al. This content is distributed under the terms of the Creative Commons Attribution 4.0 International license.

To directly compare the strains, we next performed pairwise fitness competitions between the WT and SOS variant strains (Fig. 3B). In the absence of antibiotics, there were no fitness defects observed in any competition experiments. In the presence of DNA-damaging antibiotics, the two extreme *lexA* variants, the S119A and Delta strains, had significantly reduced fitness, as expected given the MIC results. Specifically, in the presence of mitomycin C and ciprofloxacin, the wild-type strain entirely outcompeted both the S119A and Delta strains, with no colonies detected after growth. In the presence of nitrofurantoin, at the end of competition, there were no detectable colonies with the S119A strain, while the Delta strain showed a significant reduction in fitness. In contrast to the large fitness alterations observed with the S119A and Delta strains, we detected only small fitness defects for the intermediate strains relative to the WT strain, and the defect depended on the antimicrobial agent. With mitomycin C, we observed a statistically significant decrease in relative fitness for both the G80A and E86P strains. With ciprofloxacin, we observed a trend in favor of the WT over the variant strains that did not achieve statistical significance, while the levels of fitness of the G80A and E86P variants were similar to those of the WT in the presence of nitrofurantoin. Thus, we conclude that although MICs are generally unchanged for the intermediate SOS variant strains, partial reduction or enhancement of the SOS response could manifest as small changes in bacterial growth and fitness during antimicrobial stress.

### Mutagenesis under antibiotic stress.

Aside from altering antibiotic sensitivity, perturbing the SOS response has been shown to alter the likelihood of acquired antibiotic resistance (12, 34). To address the impact of SOS inhibition or activation on mutagenesis, we next applied a fluctuation analysis protocol, which utilizes rifampin selection to examine the impact of antibiotic-induced stress on the mutation rates of the strains. Unlike mutation frequencies, which can be biased by growth rates and the overall size of a population, measurement of the mutation rate by fluctuation analysis allows for an unbiased assessment of the number of mutations per generation. After bacterial growth in the presence of the stressor antibiotic, the number of mutants that acquire resistance to rifampin are counted and compared to the total CFU. The use of an orthogonal antibiotic for selection of mutants enables the estimation of the overall mutation rate of the strain under the growth conditions.

**FIG 4:**
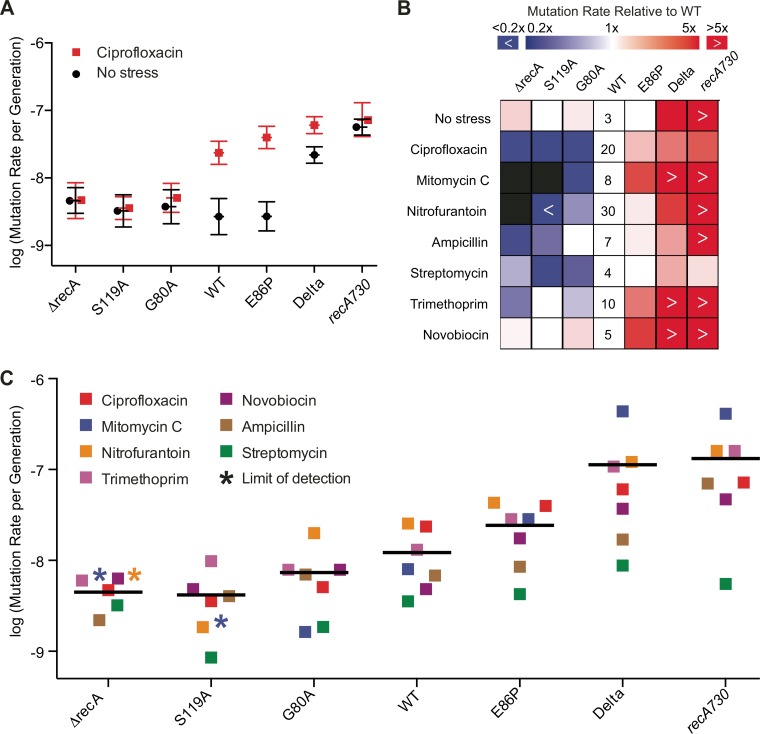
Impact of the SOS activities on *E. coli* mutation rates. (A) Mutation rates of strains grown under no stress or after exposure to sublethal doses of ciprofloxacin. Circles and squares represent the maximum likelihood mutation rate calculated from 6 replicate cultures, and the error bars represent the 95% confidence intervals. (B) Heat map of relative mutation rates across strains and antibiotic stressors. The heat map captures the reduction or increase in the mutation rate relative to the WT strain under that antibiotic. Values listed for the WT are the mutation rate (×10^−9^) per generation under each condition. Black boxes represent conditions where no resistant colonies could be detected. (C) Mutation rates of strains across a wide panel of antimicrobial agents. Data are the same as in panel B, with values shown to offer a complementary perspective for comparison between antibiotics. The squares represent individual mutation rates, and the black bars represent the mean of mutation rates under the conditions tested; error bars were removed for clarity and included in Fig. S5 in the supplemental material, with raw values listed in Table S2 in the supplemental material. Strains that did not show resistant colonies under a particular antibiotic stress are marked with an asterisk in the color of that antibiotic. The attributed values of the asterisked strain denote the mutation rate detection limit under those conditions and were excluded from the calculation of the mean mutation rate.

In the absence of antimicrobial stress, all strains displayed similar mutation rates, aside from the two constitutive SOS mutants, the Delta and *recA730* strains, which displayed approximately 7- and 20-fold more mutations per generation, respectively (Fig. 4A; see Table S2 in the supplemental material). When we introduced the noncleavable *lexA*(S119A) allele into the *recA730* strain [*recA730*(S119A)], the mutation rate was reduced back to WT levels, validating that *recA730* largely mediates mutagenesis in a LexA-dependent manner (see Fig. S5 in the supplemental material).

10.1128/mSphere.00163-16.8 Table S2  Mutation rate for drug-*E. coli* strain combinations. Download Table S2, PDF file, 0.1 MB. Copyright © 2016 Mo et al. 2016 Mo et al. This content is distributed under the terms of the Creative Commons Attribution 4.0 International license.

10.1128/mSphere.00163-16.6 Figure S5  Mutation rates of the *lexA* mutants and *recA* mutants under different types of antibiotic stress. In each plot, the stressed strains (color) are shown in comparison to the nonstressed control (black), which is the same across all conditions. Ciprofloxacin data are replicated from Fig. 4A to allow for ease of comparison. The symbol denotes the mean mutation rate, while the error bars span the 95% confidence interval derived from 6 independent measurements. Strains that did not show resistant colonies under a particular antibiotic stress are marked with an asterisk in the color of that antibiotic. The attributed value of the asterisked strain denotes the mutation rate detection limit under those conditions. Download Figure S5, PDF file, 0.5 MB. Copyright © 2016 Mo et al. 2016 Mo et al. This content is distributed under the terms of the Creative Commons Attribution 4.0 International license.

Next, we subjected the SOS variant strains to sublethal doses of ciprofloxacin. In the absence of antibiotic stress, the WT strain displays a mutation rate of 3 × 10^−9^ per replicative generation. However, in the presence of ciprofloxacin, the mutation rate increases 7-fold. Across the other strains, we observed a pattern of changes to mutation rates that correlated with the LexA cleavage rates (Fig. 4A). Mutagenesis was not induced in the S119A strain, and the mutation rate increased only 1.3-fold for the hypoactive G80A strain relative to the unstressed condition. Compared to the 7-fold induction of mutagenesis in the WT strain, we observed an approximately 13-fold increase with the hyperactive E86P variant. Notably, even in the Delta strain, we found a 3-fold enhancement of mutation rate with ciprofloxacin, suggesting that mutagenesis is enhanced by the combination of antibiotic stress with a constitutively active SOS pathway. The ciprofloxacin-induced mutation rates were similar whether *lexA* or *recA* is perturbed on the two extremes: the Δ*recA* and S119A strains showed mutation rates of 5 × 10^−9^ and 4 × 10^−9^, respectively, and the *recA730* and Delta strains displayed rates of 6 × 10^−8^ and 7 × 10^−8^, respectively.

We then examined a broader range of antibiotic stressors and found that LexA cleavage rates impact mutation rates across many different antibiotic classes. For each variant strain, we plotted the mutation rate relative to the WT (Fig. 4B). For clarity, the individual mutation rate plots are also shown in Fig. S5 and the raw values are summarized in Table S2 in the supplemental material. Across most antibiotics examined, we generally observed that SOS attenuated strains have reduced mutation rates relative to the WT, while SOS hyperactive strains have higher mutation rates. The gradation of induced mutagenesis extended to mitomycin C, nitrofurantoin, β-lactams, and trimethoprim, but was less apparent with the ribosomal inhibitor streptomycin, which can potentially impact the translation of SOS effectors, and with novobiocin, which has been reported to potentially antagonize the SOS response (48). Integrating across the series of antibiotics with different mechanisms of action, the average rate of induced mutagenesis is correlated with increased levels of SOS activation (Fig. 4C). This trend contrasts with the MIC data, where MICs tended to peak with the WT strain and were reduced when the SOS pathway was either inactive or hyperactive.

Notably, the S119A and Δ*recA* strains showed reductions in induced mutagenesis across multiple different agents beyond ciprofloxacin. With these strains, we were unable to select for rifampin-resistant mutants with mitomycin stress and observed 15-fold or greater reductions with nitrofurantoin and smaller reductions with ampicillin. This result indicates that inhibition of the SOS response could plausibly slow acquired drug resistance to multiple antimicrobial agents.

To account for the effects of the *sulA* deletion, we performed the same set of fluctuation analyses on the four *sulA*^+^ strains and compared mutation rates across strains (see Fig. S5 and Table S2 in the supplemental material). In the absence of antibiotic stress, the four strains displayed mutation rates comparable to that of the Δ*sulA* strains. While minor differences were observed in the extent of induced mutagenesis across strains, the trend of increasing mutation rate with increasing LexA cleavage rate was preserved within both the *sulA*^+^ and the Δ*sulA* background.

## DISCUSSION

Given the role of the SOS pathway in adaptation and acquired resistance to antibiotics, targeting its two major regulators—LexA and RecA—has been proposed as a viable strategy to increase bacterial sensitivity to antibiotics and combat the rise of resistance. Prior studies examining antibiotic efficacy have largely focused on the loss of normal *recA* or *lexA* function in isolation (12, 34, 49–51). These studies validated the SOS response as an interesting target and motivated our effort to explore the consequences of the various different approaches that could be taken to target LexA or RecA proteins. To this end, our study attempted to profile the full spectrum of SOS activation, including parallel comparisons of partial or complete SOS activation or repression, to help provide a road map for several key issues relevant to future inhibitor discovery efforts.

### Bacterial susceptibility to antibiotics.

Our study provides evidence that either inhibition or activation of the SOS pathway can synergize with specific antimicrobial agents to reduce MICs to various extents. With constitutive SOS inactivation, the MIC data of the S119A and Δ*recA* mutants are in agreement with previous studies: relative to the WT, both strains show enhanced sensitivity to some DNA-damaging agents, such as mitomycin C, fluoroquinolones, and nitrofurantoin (34, 52, 53). However, this increased sensitivity is largely abolished when the level of SOS induction is merely attenuated by a slow-cleaving LexA variant, the G80A strain, which shows similar MIC and only minor defects in fitness competition relative to the WT. With antimicrobials with other mechanisms of action, we see only minor changes in sensitivity relative to the WT strain, even in the SOS-inactive S119A or Δ*recA* strain. Thus, we conclude that potent inhibition of the SOS pathway in concert with particular DNA-damaging agents, including fluoroquinolones and nitrofurantoin, offers the best options for potential synergy.

Proposals to target the SOS response have largely focused on inhibiting the pathway, and to our knowledge, little data exists on how SOS overactivation affects bacterial sensitivity to antibiotics. In our study, we observed that, as with SOS-inhibited strains, strains with a constitutively active SOS response have decreased fitness relative to the wild-type strain. The reduced MICs of the Delta and *recA730* strains suggest that an overactive SOS pathway can also increase antimicrobial susceptibility, albeit to a lesser extent than observed with constitutive inhibition. DNA-damaging agents that were effectively enhanced by SOS inhibition appear to achieve the highest levels of synergy in SOS overactive strains as well, while other drugs show lesser effects. One important caveat to our result is that we were unable to experimentally examine the effects of *sulA*-mediated lethality during constitutive SOS expression, given that the Δ*lexA* and *recA730* strains require *sulA* deletion. Small molecules that disrupt LexA binding to SOS genes or activate RecA could potentially show greater synergy than we observed in the presence of *sulA*. Furthermore, even if resistance were to arise to SOS-hyperactivating agents through the deletion of *sulA*, our results with *sulA*-null strains suggest that synergy would still be anticipated to some degree.

Together our data indicate that the native function of the SOS pathway is important for bacterial adaptation to antibiotics. Both an inactivated SOS response and constitutively active SOS response can reduce bacterial viability in concert with antimicrobials that cause DNA damage. While our work does not address the mechanisms involved in altered antibiotic sensitivity, several plausible hypotheses can be considered. Because the SOS response functions primarily in DNA damage repair, perturbing the network likely prohibits bacteria from repairing the genotoxic damage caused by these agents: in the case of SOS inhibition, the DNA repair mechanisms are silenced, while with disinhibition, the processes might be uncoordinated, energetically costly, and/or promote genetic instability.

### Bacterial mutagenesis during antibiotic treatment.

Our study also informs efforts to slow mutagenesis and acquired resistance by targeting the SOS pathway. While the most significant reductions in MICs were noted at both extremes of SOS activity, the mutation rates across strains displayed a trend of continual increase, with the constitutively inactive and active SOS variants showing the lowest and highest antibiotic-induced mutation rates, respectively. Our results with constitutively inactive and active variants are consistent with more limited comparisons carried out in previous reports (12, 49, 51, 54, 55). Furthermore, we show that partial SOS pathway attenuation (G80A) or hyperactivation (E86P) results in changes to the mutation rate. Our data are also consistent with a recent report that used a mutation accumulation whole-genome sequencing approach to show that the mutation rate is strongly correlated with the dose of fluoroquinolone stress, which presumably results in different levels of SOS induction (56). We speculate that the tunable behavior for mutagenesis could be due to the regulation of the error-prone DNA polymerases, whose basal levels and stress-induced levels are likely altered by changes to LexA and RecA protein levels. Importantly, from a therapeutic angle, our data suggest that reducing LexA’s cleavage rate has the potential to attenuate mutagenesis under a range of different types of antibiotic stressors, not simply DNA-damaging antibiotics. While our study focuses on inducible mutagenesis controlled by the SOS pathway, the results also hold implications for constitutive hypermutators, which may make important clinical contributions to acquired antibiotic resistance (57). Activation of the SOS pathway may provide a mechanism for acquiring mutations that “fix” hypermutation, such as inactivating mutations in mismatch repair enzymes (58).

### Targeting LexA versus RecA.

Both RecA and LexA have been proposed as therapeutic targets to perturb SOS induction. While small molecules that target LexA have not yet been reported to our knowledge, several agents that target RecA both *in vitro* and in bacteria have been reported (59–63). Our results suggest that the effects of the SOS-inactive mutant (Δ*recA* and S119A) strains largely mimic one another in both synergy and acquired resistance, although a slightly greater degree of sensitivity is seen with the Δ*recA* strain, particularly with nitrofurantoin. Constitutive activation of the SOS pathway has similar effects in the Delta and *recA730* strains, with a slight degree of higher sensitivity seen with the Delta strain. Overall, we conclude that agents that target LexA, RecA, or their interface would all be similarly viable strategies to pursue.

Although our data suggest targeting either RecA or LexA might be of interest, it is worth noting that each likely presents its own distinctive challenges. RecA, unlike LexA, has important human homologues (Rad51 family), making specificity a particularly important requirement for targeting RecA; indeed, some high-throughput screening efforts have yielded compounds that readily target both RecA and Rad51 (64). Although protein interfaces can be challenging to target, the RecA oligomerization interface may be a valuable molecular target, particularly since RecA’s ATPase activity is thought to be nonessential for SOS activities (65). For LexA, the self-cleavage reaction of the protein’s catalytic domain makes it a difficult target for competitive inhibition, given the high local concentration of the cleavage loop “substrate” around the LexA active site. We therefore speculate that allosteric inhibitors of LexA or compounds that disrupt the LexA-RecA interface will likely offer more promise. Finally, the selection pressures for acquired resistance might also be very different for these two targets. It is reassuring that deletion of the *lexA* gene is also associated with altered sensitivity to some antibiotics, as this result implies that resistance to anti-LexA agents is less likely to arise from simple inactivating mutations.

### Strategies for targeting the SOS response.

In aggregate, our results provide a framework for developing strategies to target the SOS pathway. Regarding the question of whether to inhibit or hyperactivate the SOS response, in line with known aspects of SOS function, we suggest that inhibiting the pathway is the more feasible strategy, since it can reduce bacterial viability while also tempering stress-induced mutagenesis. Although forcing constitutive SOS induction does reduce fitness, it comes at a cost of increased mutagenesis, thus increasing the likelihood of acquired resistance. As noted, one limitation to our conclusion is that we were unable to explore the effects of SOS disinhibition in the presence of *sulA*; however, we speculate that *sulA*-dependent toxicity would likely lead to rapid inactivation of *sulA*, as its deletion does not appear to cause a strong fitness burden. With regard to the mechanism of action, our data suggest that reducing mutagenesis may offer more therapeutic opportunities with different classes of existing antibiotics. We found that numerous antibiotics can increase the mutation rate and that induced mutagenesis is decreased with loss of SOS induction. Even attenuation of the SOS pathway with the G80A strain is associated with reduced mutagenesis. While the effects on the mutation rate are modest relative to those of hypermutator strains of bacteria that lack mismatch repair, such strains with “intermediate” mutation rates may in fact be more likely to evolve multidrug resistance (66). It is also important to note that we limited our analysis to the effect on MIC and acquired resistance; however, it is possible that targeting the SOS pathway could have other benefits. In diverse species, including *Clostridium difficile*, *Vibrio cholerae*, and *Staphylococcus aureus*, the SOS pathway has been linked to pathogenic processes, including persistence, horizontal gene transfer, and expression of toxins or virulence factors (26, 27, 50, 67–69). Our data taken as a whole suggest that although many alternative strategies may be viable, the addition of an SOS inhibitor targeting RecA or LexA with a DNA-damaging antibiotic such as a fluoroquinolone could be an optimal approach to both increase susceptibility as well as decrease acquired resistance in important bacterial pathogens.

## MATERIALS AND METHODS

### Congenic strain generation.

The Δ*recA*::(FRT*-kan-*FRT) and Δ*sulA*::(FRT*-kan-*FRT) *E. coli* strains (JW3470 and JW0941, respectively) were obtained from the Keio collection (70). (In each of these strains, “FRT” represents the flippase [Flp] recombination target.) The *recA* and *sulA* genes were deleted from MG1655 using T4GT7 bacteriophage transduction (71), or P1_vir_ transduction (72), respectively, followed by Flp-mediated removal of the *kan* cassette using plasmid pCP-20 (73). The resulting Δ*recA* strain (MG1655 Δ*recA*::FRT) was subsequently used in experiments, while the Δ*sulA* strain (SAMP02 [MG1655 Δ*sulA*::FRT]) served as the parent for further genetic engineering at the *lexA* locus.

In the Δ*sulA* background of SAMP02, a Δ*lexA*::(Cm-I-SceI) cassette was introduced into the native *lexA* locus by recombineering, resulting in strain SAMP04 [MG1655 Δ*lexA*::(Cm-I-SceI) Δ*sulA*::FRT], which we refer to as the Delta strain. In brief, the chloramphenicol cassette and I-SceI restriction site were generated by PCR using the plasmid pWRG100 as the template. PCR primers have a 5′ region of homology to *lexA*, allowing for double recombination of the cassette. The Delta strain was used for recombineering-based scarless mutagenesis (74). Overlap extension was used to generate cassettes containing 1-kb regions upstream and downstream of the *lexA* gene, along with the desired *lexA* variant (the S119A, G80A, WT, and E86P strains) (75). These cassettes were transformed into the Delta strain, and counterselection yielded the desired transformants as previously described (74). These strains, which are all derivatives of the Delta strain containing the *sulA* deletion, will be referred to as the S119A, G80A, WT, and E86P strains. The *recA730* allele was introduced into the S119A and WT strains by P1_vir_ transduction using SS4247 as a donor strain followed by selection with tetracycline.

For experiments examining the effect of *sulA* deletion in our strains, we reengineered the *sulA* gene into the S119A, G80A, and E86P strains by transduction using a P1_vir_ lysate derived from MMR102, which contains Δ*torT*::(FRT*-kan-*FRT) at a locus neighboring *sulA* (72). The *kan* cassette was subsequently removed by Flp-mediated recombination using plasmid pCP20 (73) to yield strains S119A (*sulA*^+^), G80A (*sulA*^+^), and E86P (*sulA*^+^). For comparison to these *sulA^+^* strains, we used MG1655 as a reference with native *lexA*.

All strains were confirmed by PCR and sequenced at relevant loci. Strain names and relevant genotypes are summarized in Table 1. Primer sequences or strains are available upon request.

### Measuring the MIC of strains.

MICs were determined using a resazurin-based assay (76) using serial 2-fold dilutions of the drug and following previously published guidelines (77). The reported MICs represent the average of four independent measurements performed on separate days.

### Measuring strain growth rates.

Overnight cultures of the strains were diluted 10^6^-fold into fresh LB. One hundred microliters of diluted culture was distributed into a 96-well, round-bottom, transparent plate. One hundred microliters of LB with sublethal doses of antibiotic agent was then added to the cultures, and the plates were sealed with transparent tape. The cultures were incubated at 37°C with cycled agitation (3-mm orbital shaking, 450 rpm) for 16 h on a Tecan plate reader. Optical density at 595 nm (OD_595_) measurements were obtained every 30 min. We noted that these plate-based growth conditions result in an approximately 5% increase in doubling times compared to culture conditions with larger volumes in aerated 15-ml culture tubes (unpublished data). However, these conditions permit continuous measurement, and the results obtained parallel those from competition experiments (detailed below), which were performed under conditions with more significant aeration.

### Competition experiments.

For the competition experiments, a constitutively active GFP expression plasmid, pMS p*Rev-GFP* (see Text S1 in the supplemental material), was transformed into the WT strain using standard chemical transformation techniques. The same vector with constitutively repressed GFP expression (pMS p*Ara-GFP*) was transformed into the four other variant strains. Competition assay procedures were adapted from established protocols (78). In brief, overnight cultures in LB containing kanamycin for plasmid maintenance were grown. The next morning, cocultures were started by inoculating 3 ml of LB containing kanamycin with equal amounts (10^6^ dilutions) of the WT and a mutant strain in either the absence or presence of an additional antibiotic stressor. The coculture was then incubated at 37°C with aeration for 24 h. To determine the starting and final CFU of the strains, culture samples were taken before and after the 24-h growth period, plated onto LB agar (with kanamycin selection), and incubated overnight at 37°C. The next day, WT colonies on the plate were distinguished from the variant colonies by visualizing GFP fluorescence on a Bio-Rad GelDoc XR+ imager with UV illumination through an XcitaBlue conversion screen (Bio-Rad). Quantifying population numbers (*N*), we estimated the relative fitness (*W*) of the variant strain relative to the WT strain by calculating the change in the relative abundances between the initial (*i*) and final (*f*) samples (78):

$$W\;=\text{ln}(\frac{N^{f}\;\text{of variant}}{N^{i}\;\text{of variant}})\;/\;\text{ln}\;(\frac{N^{f}\;\text{of WT}}{N^{i}\;\text{of WT}})$$

When no rifampin-resistant colonies could be detected, we calculated the lower limit of detection by assuming one resistant colony was present and determining the mutation rate based on the CFU detected under those conditions. We used a two-sided paired *t* test to assess for statistical significance between the fitness of each variant strain compared to the WT strain and interpreted a *P* value of <0.05 as a significant difference.

10.1128/mSphere.00163-16.1 Text S1  Supplemental experimental procedures. Download Text S1, PDF file, 0.1 MB. Copyright © 2016 Mo et al. 2016 Mo et al. This content is distributed under the terms of the Creative Commons Attribution 4.0 International license.

### Fluctuation analysis of bacterial mutation rates.

Fluctuation analysis was performed using an adapted version of established protocols (79). Briefly, six replicate 30-ml cultures in LB (containing either no antibiotic or a sublethal level of antibiotic) were inoculated with a 3.3 × 10^8^ dilution of overnight LB broth culture. The concentration of antibiotic used in each case was a fixed level below the MIC (as provided in Table S2 in the supplemental material). The concentration of stressor antibiotic selected was the highest concentration that permitted sufficient viable CFU to allow for fluctuation analysis to be performed—typically 1.5- to 8-fold below the MIC. Cultures were grown to saturation at 37°C for 48 h. After 48 h, to determine the total CFU, appropriate dilutions were plated onto nonselective LB agar plates. To determine the number of mutant colonies, 300 µl of culture was separately spun down, washed with 100 µl of 5% NaCl solution, and plated onto 100 µg/ml of rifampin. The rifampin plates were incubated at 37°C for a total of 48 h; resistant colonies were counted once after 24 h and a second time after 48 h. The numbers of total cells and resistant colonies were then entered into the MutRateCalc software to perform maximum likelihood analysis, which yields the mutation rate and associated confidence intervals (79).
