# Supplementary figures and images for: Systematically Altering Bacterial SOS Activity under Stress Reveals Therapeutic Strategies for Potentiating Antibiotics
Source: mSphere. 2016 Aug 10;1(4):e00163-16. doi: 10.1128/mSphere.00163-16 (PMC4980697; doi:10.1128/mSphere.00163-16)

FIG S1

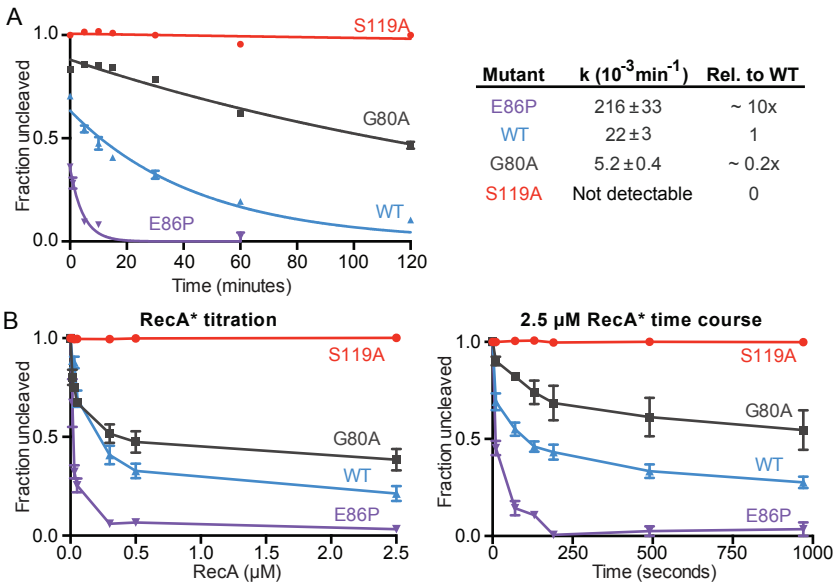

Supplement: Figure S1 [file sph004162127sf2.pdf]

FIG S2

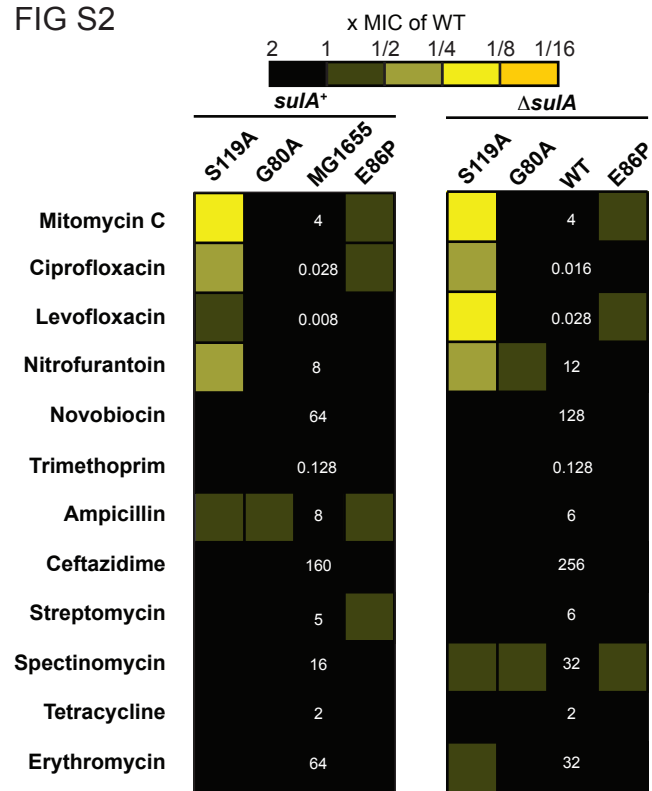

Supplement: Figure S2 [file sph004162127sf3.pdf]

FIG S3

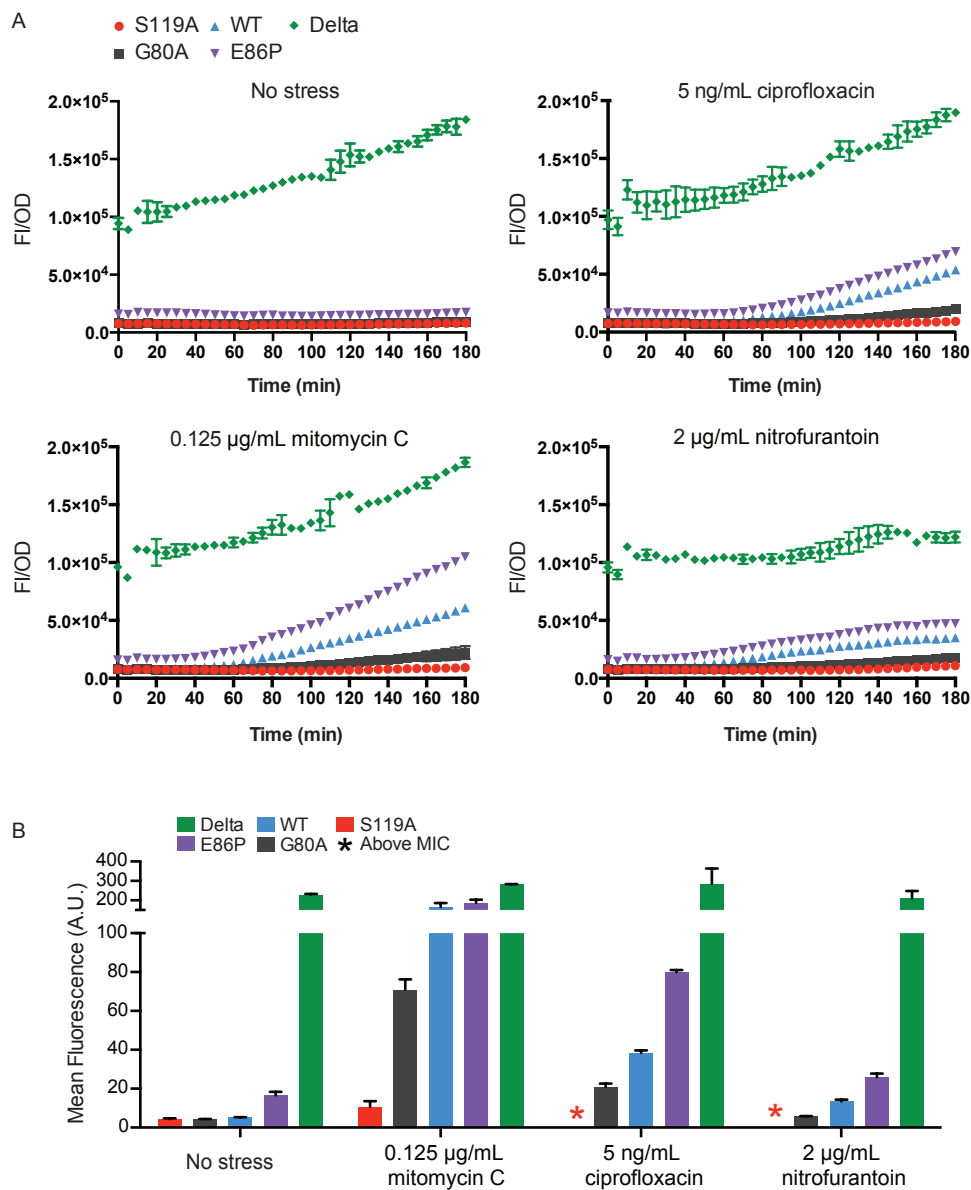

Supplement: Figure S3 [file sph004162127sf4.pdf]

FIG S4

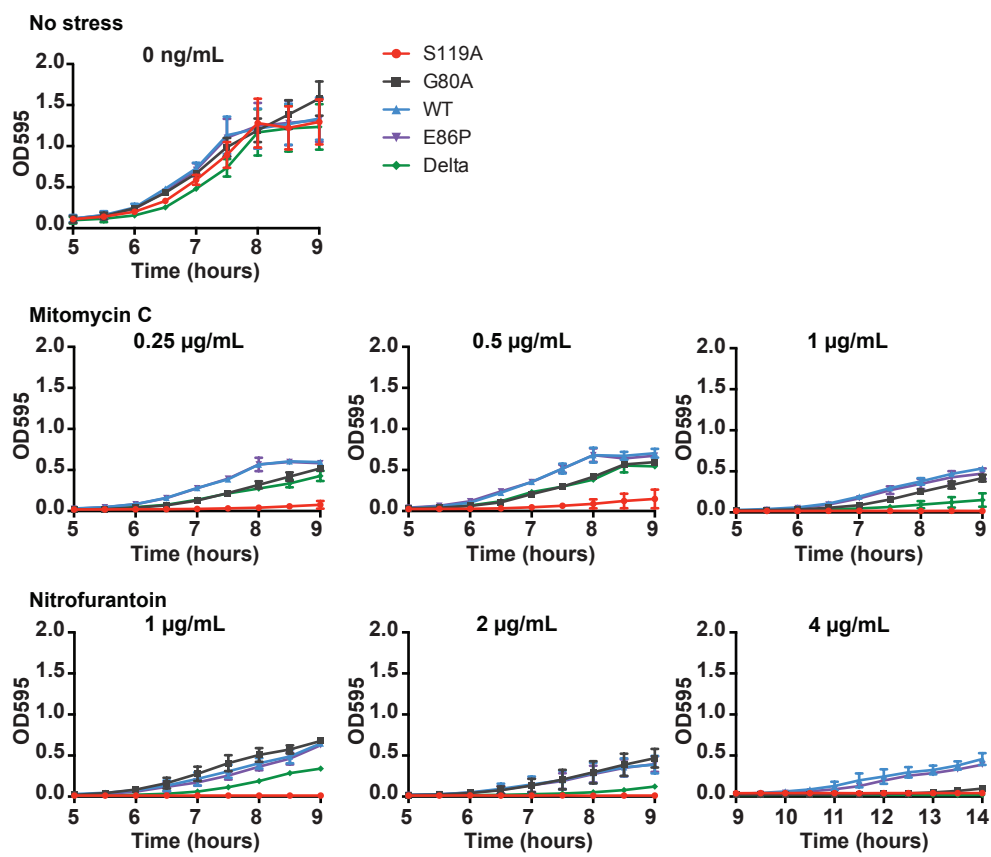

Supplement: Figure S4 [file sph004162127sf5.pdf]

FIG S5

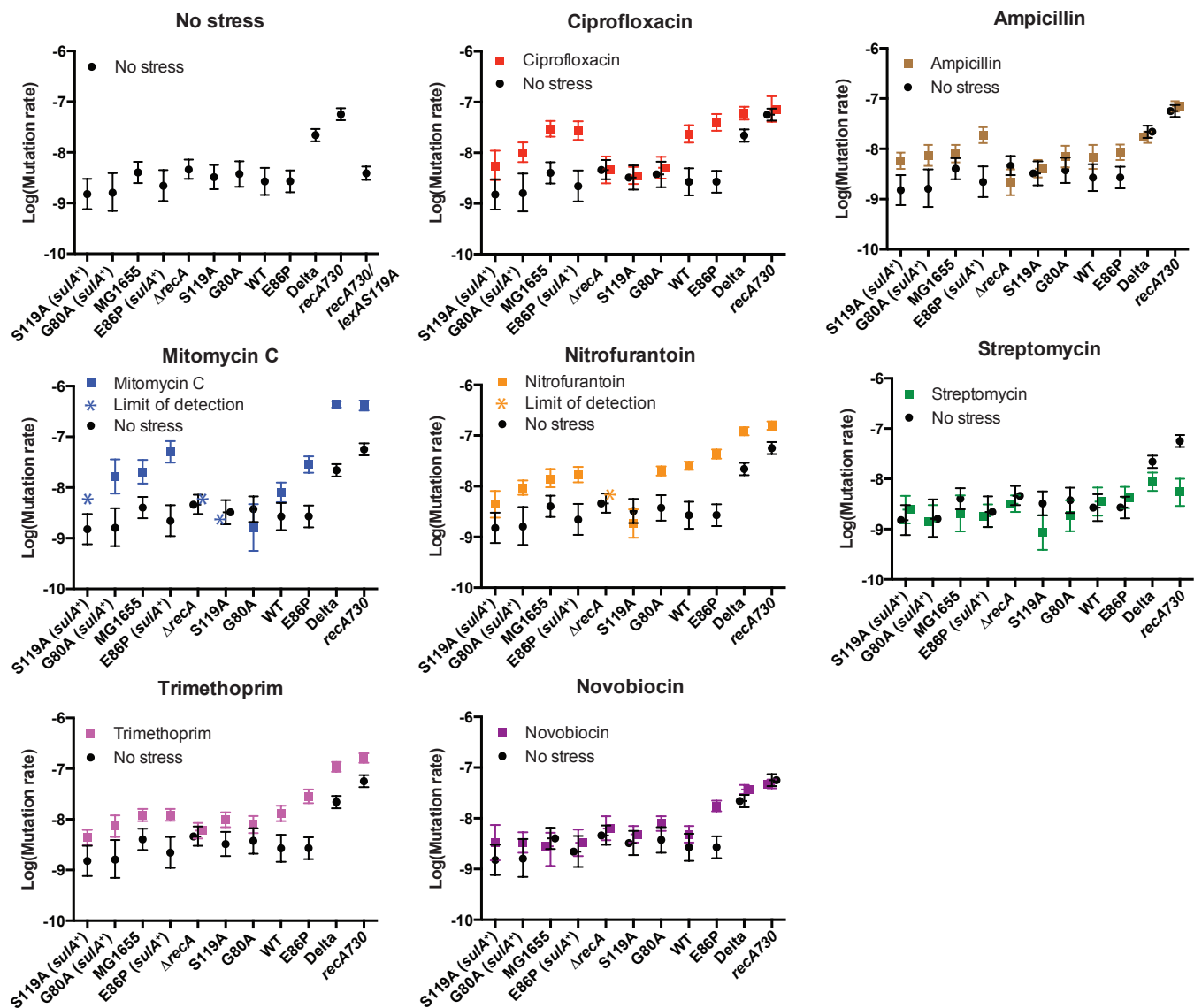

Supplement: Figure S5 [file sph004162127sf6.pdf]
