# Supplementary material for: Systematically Altering Bacterial SOS Activity under Stress Reveals Therapeutic Strategies for Potentiating Antibiotics
Source: mSphere. 2016 Aug 10;1(4):e00163-16. doi: 10.1128/mSphere.00163-16 (PMC4980697; doi:10.1128/mSphere.00163-16)
Supplement: Table S1 [file sph004162127st7.pdf]

Table S1. MICs for drug - *E. coli* strains combinations

Antibiotic (µg/mL)	Strains										
	S119A (*sulA*+)	G80A (*sulA*+)	MG1655	E86P (*sulA*+)	*ΔrecA*	S119A	G80A	WT	E86P	Delta	*recA730*
**Mitomycin C**	0.88	4	4	3	0.25	0.75	4	4	3	2	2
**Ciprofloxacin**	0.012	0.028	0.028	0.024	0.004	0.004	0.016	0.016	0.016	0.008	0.012
**Levofloxacin**	0.004	0.016	0.008	0.012	0.0018	0.006	0.028	0.028	0.016	0.007	0.014
**Nitrofurantoin**	3	8	8	10	0.375	5.5	8	12	12	8	8
**Novobiocin**	128	128	64	160	64	128	128	128	128	128	64
**Trimethoprim**	0.256	0.256	0.128	0.256	0.128	0.128	0.128	0.128	0.128	0.128	0.128
**Ampicillin**	6	6	8	6	8	7	6	6	6	4	7
**Ceftazidime**	192	224	160	224	384	288	288	256	256	160	384
**Streptomycin**	8	7	5	4	4	8	8	6	6	7	10
**Spectinomycin**	16	16	16	16	16	24	16	32	28	16	32
**Tetracycline**	2	2	2	2	1	2	2	2	2	2	ND
**Erythromycin**	64	64	64	64	20	24	32	32	32	20	32
