# Supplementary material for: Systematically Altering Bacterial SOS Activity under Stress Reveals Therapeutic Strategies for Potentiating Antibiotics
Source: mSphere. 2016 Aug 10;1(4):e00163-16. doi: 10.1128/mSphere.00163-16 (PMC4980697; doi:10.1128/mSphere.00163-16)
Supplement: Table S2 [file sph004162127st8.pdf]

Table S2. Mutation Rate for drug - *E. coli* strains combinations

	Strain	Drug (sublethal concentration, µg/mL)	Mutation rate	95% Confidence Interval
**No stress**	S119A (*sulA*$^+$)	N/A	2e-09	8e-10 – 3e-09
	G80A (*sulA*$^+$)	N/A	2e-09	7e-10 – 4e-09
	MG1655	N/A	4e-09	5e-09 – 7e-09
	E86P (*sulA*$^+$)	N/A	3e-09	1e-09 – 5e-09
	ΔrecA	N/A	5e-09	3e-09 – 7e-09
	S119A	N/A	3e-09	2e-09 – 6e-09
	G80A	N/A	4e-09	2e-09 – 7e-09
	WT	N/A	3e-09	1e-09 – 5e-09
	E86P	N/A	3e-09	2e-09 – 4e-09
	Delta	N/A	2e-08	2e-09 – 3e-08
	recA730	N/A	6e-08	4e-08 – 7e-08
	recA730/ lexAS119A	N/A	4e-09	3e-09 – 5e-09
**Ciprofloxacin**	S119A (*sulA*$^+$)	0.0025	6e-09	3e-09 – 1e-08
	G80A (*sulA*$^+$)	0.01	1e-08	7e-09 – 2e-08
	MG1655	0.01	3e-08	2e-09 – 4e-08
	E86P (*sulA*$^+$)	0.01	3e-09	2e-08 – 4e-08
	ΔrecA	0.001	5e-09	3e-09 – 9e-09
	S119A	0.0025	4e-09	2e-09 – 5e-09
	G80A	0.01	5e-09	3e-09 – 8e-09
	WT	0.01	2e-08	2e-08 – 4e-08
	E86P	0.01	4e-08	3e-08 – 6e-08
	Delta	0.005	6e-08	5e-08 – 8e-08
	recA730	0.001	7e-08	4e-08 – 1e-07
**Mitomycin C**	S119A (*sulA*$^+$)	0.125	N/A	N/A
	G80A (*sulA*$^+$)	0.25	2e-08	8e-09 – 4e-08
	MG1655	0.5	2e-08	1e-08 – 4e-08
	E86P (*sulA*$^+$)	0.5	5e-08	3e-08 – 8e-08
	ΔrecA	0.0625	N/A	N/A
	S119A	0.125	N/A	N/A
	G80A	0.25	2e-09	6e-10 – 5e-09
	WT	0.5	8e-09	5e-09 – 1e-08
	E86P	0.5	3e-08	2e-08 – 4e-08
	Delta	0.5	4e-07	4e-07 – 5e-07
	recA730	0.5	4e-07	3e-07 – 5e-07
**Nitrofurantoin**	S119A (*sulA*$^+$)	0.25	4e-09	2e-09 – 8e-09
	G80A (*sulA*$^+$)	2	9e-09	7e-09 – 1e-08
	MG1655	2	1e-08	9e-09 – 2e-08
	E86P (*sulA*$^+$)	2	2e-08	1e-08 – 2e-08
	ΔrecA	0.1	N/A	N/A
	S119A	0.5	2e-09	1e-09 – 4e-09
	G80A	4	2.0e-08	1.6e-08 – 2.5e-08
	WT	4	3.0e-08	2.0e-08 – 3.1e-08
	E86P	4	4.0e-08	3.5e-08 – 5.3e-08
	Delta	2	1.2e-07	1.0e-07 – 1.5e-07
	recA730	2	1.6e-07	1.3e-07 – 1.9e-07
**Ampicillin**	S119A (*sulA*$^+$)	2	6e-09	4e-09 – 8e-09
	G80A (*sulA*$^+$)	2	7e-09	5e-09 – 1e-08
	MG1655	2	8e-09	5e-09 – 1e-08
	E86P (*sulA*$^+$)	2	2e-08	1e-08 – 3e-08
	ΔrecA	2	2e-09	1e-09 – 4e-09
	S119A	2	4e-09	3e-09 – 6e-09
	G80A	2	7e-09	4e-09 – 1e-08
	WT	2	7e-09	4e-09 – 1e-08
	E86P	2	9e-09	6e-09 –1e-08
	Delta	2	1.7e-08	1.3e-08 – 2.2e-08
	recA730	2	7e-08	6e-08 – 9e-08

Table S2; continued from above

	Strain	Drug (sublethal concentration, µg/mL)	Mutation rate	95% Confidence Interval
**Streptomycin**	S119A (*sulA*$^{+}$)	2	2e-09	1e-09 – 5e-09
	G80A (*sulA*$^{+}$)	2	1e-09	7e-10 – 3e-09
	MG1655	2	2e-09	9e-10 – 5e-09
	E86P (*sulA*$^{+}$)	2	2e-09	1e-09 – 3e-09
	ΔrecA	2	3e-09	2e-09 – 5e-09
	S119A	2	9e-10	4e-10 – 2e-09
	G80A	2	2e-09	9e-10 – 4e-09
	WT	2	4e-09	2e-09 – 7e-09
	E86P	2	4e-09	3e-09 – 7e-09
	Delta	2	9e-09	6e-09 – 1e-08
	recA730	2	6e-09	3e-09 – 1e-08
**Trimethoprim**	S119A (*sulA*$^{+}$)	0.032	4e-09	3e-09 – 6e-09
	G80A (*sulA*$^{+}$)	0.032	7e-09	5e-09 – 1e-08
	MG1655	0.032	1e-08	9e-09 – 2e-08
	E86P (*sulA*$^{+}$)	0.032	1e-08	9e-09 – 2e-08
	ΔrecA	0.032	6e-09	4e-09 – 9e-09
	S119A	0.032	1.0e-08	7e-09 – 1.4e-08
	G80A	0.032	8e-09	5e-09 – 1e-08
	WT	0.032	1e-08	9e-09 – 2e-08
	E86P	0.032	3e-08	2e-08 – 4e-08
	Delta	0.032	1.0e-07	9e-08 – 1.4e-07
	recA730	0.032	1.6e-07	1.3e-07 – 2.0e-07
**Novobiocin**	S119A (*sulA*$^{+}$)	16	3e-09	1e-09 – 7e-09
	G80A (*sulA*$^{+}$)	16	3e-09	2e-09 – 5e-09
	MG1655	16	3e-09	1e-09 – 5e-09
	E86P (*sulA*$^{+}$)	16	3e-09	2e-09 – 6e-09
	ΔrecA	16	6e-09	4e-09 – 1.0e-08
	S119A	16	5e-09	3e-09 – 7e-09
	G80A	16	8e-09	6e-09 – 1.0e-08
	WT	16	5e-09	3e-09 – 7e-09
	E86P	16	2e-08	1.4e-08 – 2.2e-08
	Delta	16	4e-08	3e-08 – 5e-08
	recA730	16	5e-08	4e-08 – 6e-08
