# Supplementary material for: Systematically Altering Bacterial SOS Activity under Stress Reveals Therapeutic Strategies for Potentiating Antibiotics
Source: mSphere. 2016 Aug 10;1(4):e00163-16. doi: 10.1128/mSphere.00163-16 (PMC4980697; doi:10.1128/mSphere.00163-16)
Supplement: Text S1 [file sph004162127s1.pdf]

Systematically altering bacterial SOS activity under stress reveals therapeutic strategies for potentiating antibiotics

Supplemental Experimental Procedures

Generation, expression, and purification of LexA cleavage mutants. A synthetic gene encoding *E. coli* LexA was obtained from Integrated DNA Technologies and cloned it into a pET41 expression vector with a N-terminal poly-His tag and a PKA phosphorylation site. Unique restriction enzyme cut sites were positioned throughout the gene, allowing for facile cassette-based mutagenesis, as previously described (1). Using cassette mutagenesis, four LexA variants representing a spectrum of LexA cleavage rates were made: the catalytic mutant (S119A), a slow cleaver (G80A), the wild type enzyme (WT), and a fast cleaver (E86P). Following cloning, the S119A, G80A, and WT proteins were expressed and purified as previously described (1). The proteins were then further purified using size-exclusion chromatography into storage buffer (50 mM Tris-HCl, pH 7.0, 150 mM NaCl, 10% glycerol). Due to its hypercleavable nature, the E86P variant was expressed at 16 ºC overnight, rather than at 30 ºC for 4 hours, and all subsequent purification steps were carried out at 4 ºC, rather than room temperature.

Radioactive labeling of purified LexA. LexA labeling was performed on the S119A, G80A, and WT proteins for 1 hour at 30 ºC. Reactions contained 50 mM Tris-Cl, pH 7.5, 70 mM NaCl, 10 mM $MgCl_2$ and 1 μM ATP (3000 Ci [γ-^{32}P] ATP/mmol ATP) with 40000 units of PKA kinase. Unincorporated label was removed using a Microspin G50 spin column pre-equilibrated with a PIPES buffer (50 mM PIPES pH 6.5, 150 mM NaCl). Due to its hyperactive nature, we had difficulty obtaining adequate amounts of ^{32}P-labeled, full-length E86P protein. A large portion of the protein underwent self-cleavage during the labeling and subsequent purification processes. Therefore, for the subsequent cleavage assays below, unlabeled E86P protein was used at a higher concentration.

Base-mediated kinetic evaluation of LexA cleavage mutants. Purified LexA has been shown to undergo self-cleavage under elevated pH (2). We therefore examined the activities of the four LexA variants under base-mediated cleavage. 0.3 μM of ^{32}P-labeled S119A, G80A, and WT LexA, or 2.5 μM of unlabeled E86P were exposed to alkaline cleavage buffer (100 μM of Tris-Glycine-CAPS, 150 μM NaCl,

pH 10.6) and incubated at 37 ºC. Samples were taken in intervals, quenched with Laemmli buffer, and separated on 15% SDS polyacrylamide gels. The ^{32}P-labeled proteins were imaged via phosphor screen on a Typhoon imager, while the E86P was stained with Coomassie and imaged on a BioRad GelDoc. Protein bands were quantified with densitometric analysis and the fraction of full-length relative to total protein was calculated and fit to determine a first-order exponential decay rate using GraphPad Prism. The results are shown are in Fig. S1.

RecA-mediated cleavage of LexA cleavage mutants. To examine LexA self-cleavage under more physiological conditions, RecA-mediated LexA cleavage was characterized. Activated RecA (RecA*) was formed as previously described (1). For the RecA* titration, 0.3 μM of ^{32}P-labeled S119A, G80A, and WT LexA, or 2.5 μM of unlabeled E86P was incubated with variable amounts of RecA* for 30 min at room temperature. Reactions were quenched with Laemmli buffer and separated as above. For each reaction, pre-existing cleavage products, as determined from samples run without RecA*, were subtracted, and the cleavage product was calculated. For time course, the RecA* concentration was kept constant at 2.5 μM, quenched in intervals, and analyzed as above. The results are shown are in Fig. S1.

Evaluation of SOS induction levels in the *lexA* mutants. Strains were transformed with a GFP reporter plasmid containing *gfp* under the control of the *recA* promoter (pMS p*RecA-GFP*) (3). To assess SOS induction, strains with the reporter were grown in standard M9 minimal media supplemented with casamino acids overnight at 37 ºC in the presence of kanamycin selection (30 μg/mL). The next day, the strains were diluted 1:100 fold into fresh M9 media and allowed to grow at 37 ºC until an OD_{595} of 0.3. In a 96-well plate, 75 μL of the cultures were then mixed with 75 μL of media with or without the indicated antibiotic stressor (150 μL total culture volume; initial OD_{595} 0.15). To prevent evaporation, 50 μL of mineral oil (Sigma) was added to each well. The cultures were then grown at 37 ºC in a plate reader under agitation and the culture density (OD_{595}) and GFP fluorescence (Ex/Em: 485 nm/535 nm) were monitored continuously for 3 hours. The level of SOS induction was determined by taking the ratio of the fluorescence intensity and the optical density (FI/OD). To quantify the level of SOS induction in cultures growth for longer time periods, we grew strains overnight LB media with kanamycin (30 μg/mL) at 37 ºC.

Overnight cultures were diluted 10^6-fold into 200 μL of fresh LB/Kan in the presence of given levels of stressor antibiotics in a 96-well deep-well plate. The cultures were incubated at 37 ºC with agitation. After 16 hours, the cells were spun down and fixed in 200 μL of phosphate buffer saline and 4% paraformaldehyde. Fixed cells were analyzed using flow cytometry (BD FACSCalibur, Ex/Em: 488 nm/530 nm) and the mean fluorescence of 20000 cells in each condition was recorded. The results for SOS reporter expression at early and late time frames are shown in Fig. S3.

Design of a constitutively active and repressed GFP plasmid reporter. For the co-culture competition experiments, we generated two plasmid constructs to distinguish and quantify cultures: a reporter plasmid with constitutive GFP expression and a plasmid with constitutively repressed GFP expression. For the constitutive plasmid, we took a reporter plasmid containing GFP under the control of the *lexA* promoter and reversed the sequence of the *lexA*-binding box in order to prevent LexA repression, thus forming pMS p*Rev-GFP*. For the constitutively repressed plasmid, we obtained the plasmid pMS p*Ara*-GFP from the available promoter collection, which has GFP expression tightly repressed by the arabinose promoter (3). These plasmids were then transformed into and used to distinguish the strains, as described in Materials and Methods section in the main text.

References for Supplemental Experimental Procedures

1. **Mo, CY, Birdwell, LD, Kohli, RM.** 2014. Specificity determinants for autoproteolysis of LexA, a key regulator of bacterial SOS mutagenesis. Biochemistry. **53:**3158-3168. doi: 10.1021/bi500026e [doi].

2. **Little, JW.** 1984. Autodigestion of lexA and phage lambda repressors. Proc. Natl. Acad. Sci. U. S. A. **81:**1375-1379.

3. **Zaslaver, A, Bren, A, Ronen, M, Itzkovitz, S, Kikoin, I, Shavit, S, Liebermeister, W, Surette, MG, Alon, U.** 2006. A comprehensive library of fluorescent transcriptional reporters for Escherichia coli. Nat. Methods. **3:**623-628. doi: 10.1038/nmeth895.
